# Care Groups II: A Summary of the Child Survival Outcomes Achieved Using Volunteer Community Health Workers in Resource-Constrained Settings

**DOI:** 10.9745/GHSP-D-15-00052

**Published:** 2015-09-10

**Authors:** Henry Perry, Melanie Morrow, Thomas Davis, Sarah Borger, Jennifer Weiss, Mary DeCoster, Jim Ricca, Pieter Ernst

**Affiliations:** ^a^​ Johns Hopkins Bloomberg School of Public Health, Baltimore, MD, USA; ^b^​ ICF International (Maternal and Child Survival Program), Washington, DC, USA; ^c^​ Feed the Children, Oklahoma City, OK, USA; ^d^​ Food for the Hungry, Washington, DC, USA; ^e^​ Concern Worldwide/US, New York, NY, USA; ^f^​ World Relief/Mozambique, Chokwe, Mozambique.

## Abstract

The Care Group approach, described in detail in a companion paper in this journal, uses volunteers to convey health promotion messages to their neighbors. This article summarizes the available evidence on the effectiveness of the Care Group approach, drawing on articles published in the peer-reviewed literature as well as data from unpublished but publicly available project evaluations and summary analyses of these evaluations. When implemented by strong international NGOs with adequate funding, Care Groups have been remarkably effective in increasing population coverage of key child survival interventions. There is strong evidence that Care Groups can reduce childhood undernutrition and reduce the prevalence of diarrhea. Finally, evidence from multiple sources, comprising independent assessments of mortality impact, vital events collected by Care Group Volunteers themselves, and analyses using the Lives Saved Tool (LiST), that Care Groups are effective in reducing under-5 mortality. For example, the average decline in under-5 mortality, estimated using LiST, among 8 Care Group projects was 32%. In comparison, among 12 non-Care Group child survival projects, the under-5 mortality declined, on average, by an estimated 11%. Care Group projects cost in the range of US$3–$8 per beneficiary per year. The cost per life saved is in the range of $441–$3,773, and the cost per disability-adjusted life year (DALY) averted is in the range of $15–$126. The Care Group approach, when implemented as described, appears to be highly cost-effective based on internationally accepted criteria. Care Groups represent an important and promising innovative, low-cost approach to increasing the coverage of key child survival interventions in high-mortality, resource-constrained settings. Next steps include further specifying the adjustments needed in government health systems to successfully incorporate the Care Group approach, testing the feasibility of these adjustments and of the effectiveness of Care Groups in pilot programs in government health systems, and finally assessing effectiveness at scale under routine field conditions in government health programs.

## Abstract (precis)

Care Group projects resulted in high levels of healthy behavior, including use of oral rehydration therapy, bed nets, and health care services. Accordingly, under-5 mortality in Care Group areas declined by an estimated 32% compared with 11% in areas with child survival projects not using Care Groups.

## INTRODUCTION

Evidence-based interventions—those that have been shown to improve the health of resource-constrained populations under research conditions—provide the basis for much of global health programming.^1^ Rigorous evaluation of field programs that implement multiple interventions together under more routine conditions, unfortunately, has not been given sufficient attention. The need to conduct such evaluations, however, is increasingly becoming a top priority for global health.^2^

Much of the existing evidence upon which programming is based has been obtained from testing single interventions in highly controlled field settings—often referred to as efficacy studies. Assessing the effectiveness of approaches that integrate multiple interventions under more routine field conditions constitutes a logical next step in building a strong evidence base in global health programming. Furthermore, fully understanding the contextual requirement for effective implementation of the intervention(s) is essential for broader application. That is to say, under what conditions is/are the intervention(s) likely to be effective?

Community-based approaches are now recognized as one of the most important avenues for improving nutrition and reducing child mortality, particularly in high-mortality settings with weak health systems, scarce resources, and facilities that are difficult for most of the population to access.^3^ However, much of the evidence is based on efficacy studies of individual interventions rather than on testing of the delivery of multiple interventions in more typical field settings.^4^^,^^5^

Community-based approaches are one of the most important avenues for improving child health.

Care Groups, on the other hand, provide a means to implement multiple maternal and child health interventions for improving household behaviors and health care seeking by using volunteers who visit their neighbors frequently and who provide peer-to-peer counseling. In a companion article in the current issue of *Global Health: Science and Practice*, we described this approach, providing details on what they are, how they emerged as a service delivery strategy, and the field experience with using this particular strategy.^6^ The purpose of this article is to summarize the evidence regarding the effectiveness, cost, and cost-effectiveness of the Care Group approach to improving child survival, all of which has been generated in relatively routine field conditions, and then attempt to provide further specification of the “secret sauce” that makes this approach as effective as the available evidence indicates that it is.

## METHODS

We undertook a review of the evaluations of Care Group child survival projects, which included unpublished project evaluations, presentations about Care Group projects given at global health conferences, and peer-reviewed publications. In addition, we also reviewed the findings from 2 Technical Advisory Group (TAG) meetings held in 2010 and 2014. These TAG meetings provide the opportunity for those engaged with Care Group child survival and child nutrition project implementation to review the progress, achievements, and limitations of the Care Group approach.

All but one of the early Care Group projects were funded by the United States Agency for International Development (USAID) Child Survival and Health Grants Program. These grantee projects were required to conduct household surveys to collect baseline measurements of population coverage of key interventions as well as end-of-project measures using similar survey instruments. Thus, these baseline and endline surveys provide a way to assess changes in practice and coverage over the course of the projects.

## FINDINGS

### Initial Evidence of Effectiveness: Pre/Post-Analysis of Individual Care Group Projects

Early evidence of effectiveness of the Care Group approach arose in the late 1990s and early 2000s from comparisons of baseline with end-of-project measures of population coverage of key interventions. The international NGO World Relief carried out a retrospective assessment with researchers from Johns Hopkins University of the mortality impact of its Care Group child survival project in Mozambique.^7^ This project had been implemented between 1999 and 2003 in a population of 130,000 people living in rural villages of Chokwe District. Interviewers with experience in collecting data for the Mozambique Demographic and Health Survey (DHS) were hired to obtain pregnancy histories from a representative sample of women in the project area using the standard DHS retrospective complete birth history questionnaire for measuring mortality in children younger than 5 years of age. The independent mortality assessment demonstrated that the under-5 mortality rate had declined by 44.2%, from 163 per 1,000 births (95% confidence interval [CI] = 130, 230) to 91 per 1,000 births (95% CI = 57, 124) over the course of the project.

These findings were supported by marked increases in population coverage of key maternal and child interventions and increases in health care utilization. For example, the percentage of children with diarrhea who were treated with oral rehydration therapy increased from 53% (95% CI = 43.9, 62.1) to 94% (95% CI = 89.6, 98.4); the percentage of children who slept under an insecticide-treated bed net increased from 0% to 85% (95% CI = 80.5, 89.5); and the percentage of children with fast or difficult breathing treated at a health center/post increased from 2.0% (95% CI = 1.9, 5.9) to 60.0% (95% CI = 35.2, 84.8). Furthermore, vital events registration data collected by the Care Group Volunteers themselves indicated a decline in under-5 mortality of 62.2%, from 119 per 1,000 births (95% CI = 110, 128) to 45 per 1,000 births (95% CI = 40, 50). Using the Lives Saved Tool (LiST), which estimates mortality impact based on change in population coverage of evidence-based maternal and child health interventions, the estimated decline in under-5 mortality was 34%, very similar to the 38% decline estimated from the DHS birth history.^8^ This overall decline represents an annual decline in under-5 mortality of 9.5% per year during the life of the project compared with an annual average rate of decline (based on DHS data) of 3.0% in Gaza Province (the province where the project was located) and 4.6% per year nationally, based on DHS data.^9^^,^^10^ There were no other occurrences taking place during the project area during this time that could have produced the marked changes in coverage of key maternal and child health interventions.

World Relief conducted another Care Group project in Kampong Cham province in Cambodia from 2000–2004, which showed a similar dramatic decline in under-5 mortality of 71% according to vital events data collected by the Care Group Volunteers. In comparison, the ongoing secular decline in the province during the same period was 42%.^11^ The mortality decline in Kampong Cham province was also accompanied by marked increases in population coverage of key maternal and child health interventions and in use of health care services.^12^

World Relief later developed and implemented similarly successful Care Group projects in Malawi and Rwanda, which achieved high levels of coverage of key interventions.^13^^,^^14^ Other NGOs started to try the Care Group approach, most notably Food for the Hungry in the Sofala Province of Mozambique in 1997 and Curamericas Global in Huehuetenango, Guatemala, in 2002. Additional international NGOs, with funding from the USAID Child Survival and Health Grants Program, adopted the Care Group approach soon thereafter: American Red Cross in Cambodia, Plan International in Kenya, the Salvation Army World Service Office in Zambia, Concern Worldwide in Burundi, Medical Teams International in Liberia, and Catholic Relief Services in Malawi.

### A Growing Evidence Base: Comparison of Effectiveness Across Care Group Projects

NGOs using the Care Group approach were achieving remarkable increases in population coverage of key maternal and child health interventions in their project areas. This became more apparent when outcomes were directly compared between different projects supported by the USAID Child Survival and Health Grants Program that were using this particular service delivery strategy and undergoing similar approaches to evaluation.^15^ The comparison was done by using an early and subsequent versions of what is known today as the LiST^16^ to estimate mortality impact indirectly based on changes in population coverage of evidence-based interventions. Among 13 such projects, the estimated decline in the under-5 mortality rate ranged between 12% and 48%, with more than half of the projects achieving mortality declines above 30% (Figure).

**FIGURE:**
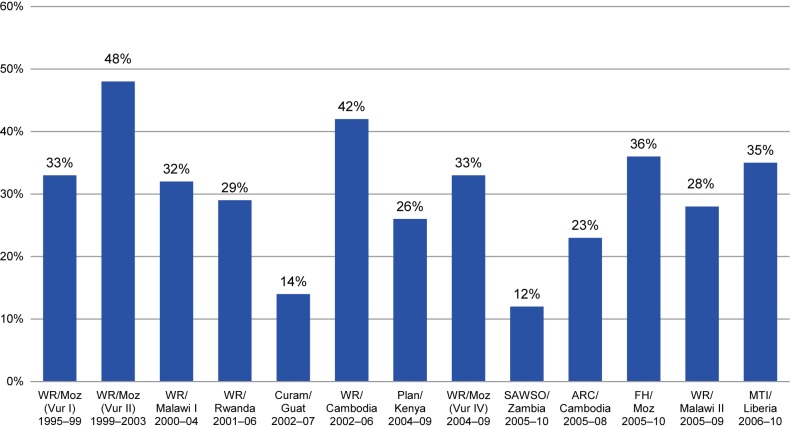
Estimated Decline in the Under-5 Mortality Rate^a^ Among 13 Care Group Projects in 8 Countries, 1995–2010 Abbreviations: ARC, American Red Cross; Curam, Curamericas; FH, Food for the Hungry; Guat, Guatemala; Moz, Mozambique; MTI, Medical Teams International; SAWSO, Salvation Army World Service Office; Vur, *Vurhonga*; WR, World Relief. Projects are listed in chronological order of initiation (from left to right). ^a^ Based on the Lives Saved Tool (LiST).^15^

Detailed data are available for 8 of these Care Group projects that had been implemented by 3 different international NGOs during the period from 1995–2010, allowing for more in-depth analysis (Table 1). Among these 8 Care Group projects, the average decline in under-5 mortality, estimated using LiST, was 32%. For a crude comparison, we can look to a separate analysis of 13 Child Survival and Health Program projects supported by USAID—only 1 of which was a Care Group project—that ended between June 2004 and June 2005.^17^ That analysis estimated (using the version of LiST current at that time) a decline in under-5 mortality of 13% among the 13 child survival projects. This comparison (32% mortality reduction among Care Group projects versus 13% reduction among general child survival projects) is only suggestive, not conclusive, of a stronger mortality impact of the Care Group approach since the comparison group does not include projects ending during the same time period as the Care Group projects, and the comparison group also includes 1 Care Group project, making it an “impure” comparison. When this Care Group project is removed from the analysis, the estimated decline in mortality is 11% for the remaining 12 non-Care Group projects (personal communication with James Ricca, first author of the original analysis of the 13 child survival projects,^17^ July 2015).

Under-5 mortality declined by an estimated 32% in areas with Care Group projects.

**TABLE 1:** Characteristics and Cost-Effectiveness of 8 Care Group Projects^a^

Care Group Project	Estimated Percentage Reduction in Under-5 Mortality^b^	No. of Beneficiaries^c^	Total Project Cost (US$)^c^	Average Cost per Beneficiary per Year	Estimated No. of Lives Saved	Cost per Life Saved	Cost per DALY Averted
World Relief/ Mozambique, *Vurhonga* I (1995–1999)	33%	57,277	$1,811,895	$7.91	819	$2,212	$27.30
World Relief/ Mozambique, *Vurhonga* II (1999–2003)	48%	53,418	$1,397,531	$6.54	769	$1,817	$60.57
World Relief/Malawi I (2000–2004)	32%	68,917	$1,333,335	$4.84	557	$2,394	$79.80
World Relief/Rwanda (2001–2006)	29%	54,451	$1,733,333	$6.37	676	$2,564	$85.47
Plan/Kenya (2004–2009)	26%	110,735	$2,300,000	$4.15	826	$2,785	$92.82
World Relief/ Mozambique, *Vurhonga* IV (2004–2009)	33%	101,757	$2,000,000	$6.56	1,217	$1,643	$54.77
World Relief/Malawi II (2005–2009)	28%	72,226	$2,022,034	$7.00	537	$3,773	$125.77
FH/Mozambique (2005–2010)	30% overall (32% in Area A; 26% in Area B)	219,617	$3,024,166	$2.78	6,848	$441	$14.72
Average of 8 Care Group projects above	32%	92,300	$1,956,016	$5.77	1,531	$2,204	$67.65
Average of 13 recent USAID-supported child survival projects^e^	13%						

Abbreviations: DALY, disability-adjusted life year; FH, Food for the Hungry; LiST, Lives Saved Tool; USAID, United States Agency for International Development.

^a^​ Source of data for the 8 Care Group projects: project final evaluations and personal communication with World Relief, Food for the Hungry, and Plan International child survival staff.

^b^​ Based on calculations using the LiST tool, uncorrected for underlying secular trends.^15^

^c^​ Number of women of reproductive age and children 0–59 months of age served by the project.

^d^​ USAID expenses plus matching funds provided by the implementing NGO.

^e^​ Ricca, 2005.^17^

Food for the Hungry, another international NGO, implemented a Care Group child survival project funded by the USAID Child Survival and Health Grants Program in 7 districts of Sofala Province in Mozambique between 2005 and 2010 in a total population of 1.1 million people. As shown in Table 2, the project achieved an annual decline in the percentage of undernourished children that was approximately 4 times greater than the underlying secular decline (2.2% versus 0.6%, respectively).^18^ The results were accompanied by major increases in the coverage of key child survival interventions related to nutrition (such as rates of exclusive breastfeeding during the first 6 months of life, frequent feeding after 6 months of age, provision of vitamin-A rich and oily foods after 6 months of age, feeding after childhood illness, and vitamin A supplementation), as well as by increased coverage of interventions to prevent and treat diarrhea (such as treatment of drinking water, hand washing, knowledge of how to prepare oral rehydration solution [ORS], and administration of ORS to children with diarrhea).

**TABLE 2:** Average Annual Rate of Decline in Undernutrition in Care Group Mozambique Project Areas Compared With Mozambique Nationwide, 2006–2010

Location	% of Children <2 SD Below the Standard Median Weight-for-Age Score		Difference	No. of Years Between Endline and Baseline	Average Annual Rate of Decline
	Baseline (Dates)	Endline (Dates)			
Project areas	26.5% (2006)	16.7% (2010)	9.8 percentage points	4.4	2.2%
Nationwide	20.0% (2003)	14.9% (2011)	5.1 percentage points	8	0.6%

Abbreviation: SD, standard deviations.

Source of data: Davis, 2013.^18^

More recently, the Care Group approach was used in a randomized controlled trial to assess the effectiveness of a behavioral change communication (BCC) intervention in reducing diarrheal prevalence in a peri-urban setting on the outskirts of Cochabamba, Bolivia.^19^ Care Groups were randomly assigned to 1 of 4 interventions: (1) the use of a special water filter (Sawyer PointONE) *without* BCC, (2) a special water filter *with* BCC, (3) BCC *without* the special water filter, and (4) a control arm in which Care Groups were used to promote an intervention unrelated to diarrhea prevention (weekly messages on life skills and attitudes such as household budgeting, valuing children, and environmental stewardship). Over a 6-month period, the 2-week prevalence of diarrhea remained in the range of 40% to 60% in the control arm while in both the Care Group BCC arm and in the Care Group BCC + water filter arm, the prevalence of diarrhea declined to one-fourth of baseline levels (9% to 14%, depending on the arm). The Care Group BCC intervention was as effective as the special water filter intervention alone and as the Care Group BCC + water filter intervention.

Using LiST, a recent analysis has compared the estimated mortality impact of 10 Care Group projects with 7 non-Care Group projects implemented in the same countries. All these projects were funded by the USAID Child Survival and Health Grants Program and were carried out between 1998 and 2010. The Care Group projects demonstrated an average annual rate of reduction in under-5 mortality that was 1.5 times greater than the rate among the non-Care Group projects (4.8% versus 3.1%, respectively).^20^ Overall, the Care Group projects yielded higher increases than the non-Care Group projects in population coverage of all 17 indicators for high-impact interventions. For example, the Care Group projects had more than twice the increase in population coverage compared with non-Care Group projects for antenatal care visits, tetanus toxoid vaccination, multiple micronutrient supplementation, complementary feeding, hand washing with soap, use of ORS to treat diarrhea, use of oral antibiotics to treat pneumonia, and malaria treatment.

An analysis of Care Group vs. non-Care Group projects found the average rate of decline in under-5 mortality was 1.5 times greater for the Care Group projects.

These findings take on additional significance because child survival projects funded by the USAID Child Survival and Health Grants Program (both Care Group and non-Care Group projects) have a documented track record of accelerating under-5 mortality reduction within their project areas over the duration of the projects. Estimates of under-5 mortality impact (using LiST) of 12 of these projects that ended between 2006–2007 have been compared with changes in under-5 mortality measured by DHS in the same countries or regions of those countries.^21^ In these countries, there was a national DHS finished within 3 years of project *initiation* and also a DHS finished within 3 years of project *completion*. The analysis demonstrated that the estimated annual under-5 mortality decline for the USAID-funded child survival projects was twice as great as the underlying secular trend in under-5 mortality decline (5.8% versus 2.5%, respectively) across a variety of settings. The results can be thought of as the “typical” results of the projects funded through this mechanism.

As part of the end-of-project evaluations of the Care Group projects, qualitative analyses were carried out in the form of key informant interviews and focus group discussions with project staff and other respondents typically including project beneficiaries, community leaders, and ministry of health (MOH) staff. Care Group Volunteers and beneficiaries have uniformly indicated that Care Groups are an effective delivery mechanism for child survival interventions. Care Groups are also empowering to the Care Group Volunteers. Many of these volunteers go on to leadership positions in their communities and beyond.

Care Groups also empower the volunteer health workers.

The great majority of Care Group projects that have been implemented so far have independently conducted end-of-project evaluations led by external evaluation consultants. A list of Care Group projects and their final project evaluation reports are publicly available.^22^ They all show marked increases in population coverage of key interventions and strongly positive assessments by project beneficiaries, community leaders, Care Group Volunteers, and project staff.

### Cost and Cost-Effectiveness of Care Group Projects

Costs of the initial Care Group projects, which were mostly funded by the USAID Child Survival and Health Grants Program, are known. This, along with the availability of LiST to estimate the number of lives saved according to the change in coverage of key child survival interventions, makes it possible to compute a cost per life saved and a cost per disability-adjusted life year (DALY) averted. Table 1 provides this information for 8 of the early Care Group projects completed in 2010 or earlier.

The average cost per beneficiary (mothers and children 0–59 months of age) per year is in the range of US$3–$8, which translates to approximately $1–$3 per population of all age groups. So, for example, the annual average cost of the Food for the Hungry/Mozambique Care Group Project was $600,000 for a population of 1.1 million people, or $0.54 per capita (for all age groups) per year. This is less than 1% of the $86 per capita recommended for spending on health services by all countries, recognizing that the poorest countries will need external support to achieve this goal.^23^ (The recommendation is by the Working Group on Health Financing in the Centre on Global Health Security at Chatham House.)

Care Group projects cost, on average, US$1–$3 per capita.

The cost per life saved (as estimated by LiST) is in the range of $441 to $3,773, and the cost per DALY averted (again, using LiST and assuming that 30 DALYs were gained for each averted death of an under-5 child) is in the range of $15 to $126 (Table 1). It should be noted that the cost per DALY gives a conservative estimate, as it does not include any measure of morbidity improvement. The accepted international standard established by the World Health Organization for a highly cost-effective intervention is a cost per DALY averted of less than the per capita gross domestic product (GDP) for the country or region where the intervention is implemented.^24^ The per capita GDP for least developed countries (where almost all Care Group projects have been implemented) is in the range of US$848 to $2,046—far above the cost per DALY averted range of $15 to $126 for Care Groups.^25^

Few studies of the cost-effectiveness of integrated community-based child survival projects and programs have been published, so comparing these findings with other approaches is a challenge. Table 3 compares cost-effectiveness data for Care Group child survival projects with data from a comprehensive primary health care program in Bolivia,^26^ a comprehensive primary health care and hospital program in Haiti,^27^ a hypothetical package of key community-based interventions,^28^ and Participatory Learning and Action (PLA) groups.^29^^,^^30^ In terms of cost per life saved and cost per DALY averted, the cost-effectiveness of Care Group projects compares favorably with that of other approaches for which mortality impact and costs have been measured or estimated.

### Limitations of the Evidence

The evidence presented here has definite limitations. Some of the data are unpublished in the peer-reviewed literature. Even so, the previously unpublished data that have been presented in this paper have been collected in a rigorous fashion. The data were derived from evaluations of USAID Child Survival and Health Grants Program child survival projects and are widely known to be of high quality. The evaluations were carried out under guidelines established by USAID Child Survival and Health Grants Program, which used accepted scientific criteria for indicator definition and measurement of population coverage. The guidelines for indicator measurement and analysis followed many of the standards established by the DHS.

There are surprisingly limited comparative data on the mortality impact and costs of integrated, community-based child survival programs. Thus, the evidence base for Care Group effectiveness, although arising from many sources and using many different criteria of effectiveness, is not as strong as it could be. This is in part because data are lacking in other quarters against which to benchmark these results. Nevertheless, the evidence is important and merits reporting in the peer-reviewed literature as a comparison for further analyses of existing data and for future studies of Care Group effectiveness, which are definitely warranted in our view.

## DISCUSSION

### What Is Required for Care Groups to Be Effective?

The Care Group approach as implemented thus far encompasses several elements, such as a certain number of households under the responsibility of each Care Group Volunteer and a certain number of Care Group Volunteers per Care Group. (See companion article in this issue of *Global Health: Science and Practice* for implementation details about the Care Group approach.^6^)

Are there specific aspects of the Care Group approach that account for its effectiveness, or is it the net sum of the various elements of the Care Group approach rather than any single element that makes Care Groups effective? This is a question that is not readily answered; no firm data exist to support specific hypotheses. However, plausible essential ingredients required for Care Group effectiveness that have been given by those with experience with Care Group implementation include:

- Identification of all target households and delivery of health education to all households
- Peer-support counseling and modeling of key behaviors by volunteer women selected by their peers (who are more likely to be “hubs” in their social networks), resulting in community-wide uptake of new behaviors and changes in community norms
- Well-designed lessons provided in small “drips” along with visual aids such as flip charts to assist the low-literacy Care Group Volunteers in sharing key practices (or behaviors) with their neighbors
- The iterative empowering nature of Care Groups, which meet every 2–4 weeks and support individual Care Group Volunteers to learn progressively how to effectively promote change with those in their catchment areas (and how to review deaths and what could be done to prevent future deaths)
- The social support the Care Group Volunteers provide to each other that is motivating and provides positive social pressure to do things well
- A combination of some or all of the above, or perhaps even a synergistic effect of some or all of the above

Peer-to-peer counseling and modeling of key behaviors is a key ingredient of the Care Group approach.

Exploring the relative importance of these elements is basically “virgin territory” for research regarding how and why community-based programming is effective (or not). A call has recently gone out for a better understanding of the mechanisms that account for the effectiveness of participatory women’s groups.^31^

Regarding the second plausible reason listed above (peer-support counseling and modeling of key behaviors), a recent groundbreaking cluster randomized controlled trial compared how different methods of targeting of potential influencers in communities affected, in turn, the influencers promoting 2 health behaviors—chlorine for water purification and multivitamins to prevent micronutrient deficiencies—to their neighbors.^32^ Villages were randomized (separately for each intervention) to 1 of 3 targeting methods, introducing the interventions to samples of either: (1) randomly selected villagers, (2) villagers with the most social ties (i.e., the largest “hubs” in the social network), or (3) “nominated friends” of random villagers (i.e., “minor hubs” in the social network). Targeting nominated friends (i.e., minor hubs in the social network) led to a 12.2% increase in adoption of the nutritional intervention compared with random targeting (95% CI = 6.9, 17.9) while targeting the *most* highly connected individuals (i.e., the major hubs in the social network) produced no greater adoption of either intervention compared with random targeting. This may be relevant to explaining the available evidence of Care Group effectiveness since Care Group Volunteers are nominated and chosen by a group of about 12 of their neighbors. These volunteers are similar to the “minor hubs.”

A “realist synthesis” of the available evidence regarding the effectiveness of the Care Group approach might be useful as a further analysis. This type of synthesis would involve “accounting for context as well as outcomes in the process of systematically and transparently synthesizing relevant literature,”^33^ with a focus on understanding the mechanisms by which an intervention works (or not).^34^ Some of the contextual features of Care Group implementation that seem important but have not been mentioned above include the following:

- The extensive amount of time spent by the Care Group Volunteer with each beneficiary mother month by month
- The engagement of beneficiaries in the selection of Care Group Volunteers
- The organization of beneficiaries into small, interactive groups that meet often and have close linkages with community leaders and health facility staff
- The minimal workloads of Care Group Volunteers (usually 3–4 hours per week) that avoid overburdening them and that enables them to perform their assigned tasks well
- The conduct of rapid formative research to select key behaviors and their determinants and to develop educational messages based on this research

Care Group Volunteers are given minimal workloads (usually 3–4 hours a week) to avoid overburdening them.

We are not aware of any studies that measure the amount of time a community-level worker spends on average with each woman in the project. For one Care Group project (the Food for the Hungry Care Group Project in Mozambique^18^), we have estimated that each beneficiary mother spent 3.3 hours per month with her Care Group Volunteer (personal communication with T. Davis, Senior Director of Program Quality Improvement, Food for the Hungry, July 2010). We think this is a reasonable estimate for other Care Group projects as well.

It is unusual for other community health worker (CHW) programs to have a case load of only 10–12 households and for the CHW to regularly visit each household—and to visit each household twice a month. Most other programs make routine visits once a month at the most to a larger number of households. In some cases, this is possible to achieve, such as in Mali where CHWs working with the United Nations Children’s Fund’s (UNICEF’s) Accelerated Strategy of Child Survival and Development are responsible for 35 households,^35^ and in Nepal where Female Community Health Volunteers are responsible for 50 households,^36^ and in Brazil where Community Health Agents are responsible for 150 households.^37^ However, in many cases it just is not practical or possible for a CHW to visit each household in his/her catchment area each month. For instance, in Eritrea, Kenya, Mozambique, and Zimbabwe, CHWs provide community case management for up to 500 households, and in Ethiopia, Malawi, and Zambia for up to 1,000 households.^38^^,^^39^

Such an analysis goes beyond the scope of this paper but could serve as a fruitful approach to better appreciate the conditions and contexts that contributed to the effectiveness of the Care Group approach as well as the conditions and contexts that would be needed in order to achieve effectiveness of the Care Group approach if implemented by MOHs at scale. The specification of elements of the Care Group approach that we think are important in explaining effectiveness, as described above, is a beginning attempt in this direction.

### Should Care Groups Be Incorporated Into Government Health Programs?

In spite of the impressive accumulated evidence regarding the effectiveness of Care Groups as a community-based intervention delivery system, the projects employing this system have all ended, unfortunately, once external donor support ended. However, NGOs are using new sources of funding—from DFID to the World Bank—to implement Care Group projects, and national NGOs are beginning to implement the approach as well. Furthermore, there is considerable anecdotal evidence as well as evidence from a follow-up survey in one project that Care Group Volunteers remain active by meeting as a Care Group and visiting the homes in their catchment area for at least several years following the end of external funding. Nonetheless, there has not yet emerged a clear approach to implementing and sustaining the Care Group approach in MOH delivery systems. This is because effective Care Group implementation requires a small number of well-trained and well-supervised Facilitators/Promoters who can focus their attention to working with Care Group Volunteers, and so far no MOH has dedicated any of its peripheral staff to carry out this task exclusively on a full-time basis.

Before MOHs are willing to do this, the current evidence regarding Care Group effectiveness will have to be disseminated, endorsement from global policy influencers will be needed, and more evidence may be necessary. Some think that that evidence will need to be in the form of controlled trials. Each controlled trial would have to determine how many interventions to include. Another school of thought is that Care Groups have proven that they are an effective vehicle for lifesaving interventions and that the basic effectiveness question has been answered. Therefore, new evidence needs to focus on how to maximize the use of this vehicle and/or on how to combine its organizing processes with other similar proven approaches such as PLA women’s groups. MOHs may be more interested in processes for packaging sets of community-based health interventions defined in their context and testing how far this integration can go, with emphasis on equity and sustainability of the model. In addition, operations research projects modeled after the Concern Worldwide/Burundi project,^40^ described in the companion paper in this series,^6^ will be needed to document how the Care Group approach might be integrated effectively and sustainably into existing MOH systems through various measures (including partnerships and contracting). In addition to meeting the requirements for effective Care Group implementation specified earlier in this paper and of having MOH staff members serve as Care Group Facilitators/Promoters, an effective Care Group project would need to have at a minimum a tight supervisory structure, highly motivated staff, and transport support to enable the staff to interact with each other and with the Care Groups.

### Next Steps

The Care Group approach, as implemented by strong international NGOs with adequate funding and in collaboration with the MOH and existing health services, has achieved an impressive record of success in terms of enthusiasm for the approach among implementers and beneficiaries as well as in terms of effectiveness (in expanding coverage of key child survival interventions, in mortality impact, and in cost-effectiveness). Unfortunately, the experiences and evidence have not yet been widely disseminated and are not well-known. The current evidence of effectiveness is sufficiently robust to justify: (1) further rigorous evaluations of the Care Group approach as implemented by NGOs in collaboration with government health programs, (2) further specification of the leadership, management, and support functions needed to implement the Care Group approach within government programs, (3) testing the effectiveness of the Care Group approach when implemented by government health programs on a pilot basis, and, assuming the results are sufficiently promising, (4) implementing and rigorously assessing the effectiveness of the Care Group approach at scale under routine field conditions in government health programs.

The minimum requirements and necessary conditions required for effective functioning of the Care Group approach in government health systems need to be defined. We have made a first attempt at this earlier in this paper and in the companion paper of this 2-part series. If the Care Group approach can be successfully integrated into government health systems, it will be important to test the effectiveness of the approach at scale. Experience with implementing the Care Group approach in urban slum settings is also needed. Given the expertise that NGOs have developed in working effectively with communities, it might turn out to be the case that long-term public-private partnerships between MOHs and NGOs may be essential for achieving sustainable effectiveness of the Care Group approach at scale.

Care Groups are not the only possible approach conceivable for educating and empowering women to adopt healthy behaviors. However, the Care Group approach is a simple and practical approach for reaching all targeted households with health promotion messages that takes advantage of peer-to-peer counseling—important elements, we believe, for strengthening the population coverage and overall effectiveness of maternal and child health programs. We encourage more experience and evaluation not only of the Care Group approach but also of other similar community-based approaches that use women’s groups in an empowering way. Over time, with more experience and rigorous evaluations of these types of approaches, stronger programs that are cost-effective will emerge.

## CONCLUSIONS

When implemented by strong international NGOs with adequate funding, Care Groups appear to be a promising approach for expanding coverage of key maternal and child interventions and for accelerating reductions in under-5 mortality and potentially maternal mortality as well—at a per capita cost of less than 4% of the current recommendation for what countries should be spending for health care services. The approach also has great potential for controlling HIV, tuberculosis, and malaria. Since the Care Group approach has been applied by many different organizations in a wide variety of settings across the world, the field experience is now extensive, and evidence of effectiveness is accumulating. More rigorous testing of the Care Group approach is now needed, as implemented by NGOs and also as implemented by government health programs. The conditions needed for Care Group effectiveness need further specification, and the Care Group approach should be implemented within government health systems on a small pilot scale to assess their feasibility and effectiveness and then, if promising, tested at scale. Assessing different ways of engaging NGOs in the process of government implementation may prove important as well.

## Notes

**Peer Reviewed**
